# The adaptation strategies of *Herpetospermum pedunculosum* (Ser.) Baill at altitude gradient of the Tibetan plateau by physiological and metabolomic methods

**DOI:** 10.1186/s12864-019-5778-y

**Published:** 2019-06-03

**Authors:** Yong Zhao, Fuling Xu, Jia Liu, Fachun Guan, Hong Quan, Fanjuan Meng

**Affiliations:** 1 College of Life Science, Northeast Forestry University, Harbin, 150040 China; 2 Key Laboratory of Plant Ecology, Northeast Forestry University, Harbin, 150040 China; 3 Jilin Academy of Agricultural Science, Changchun, 130033 China; 4 Tibet Agriculture and Animal Husbandry College, Nyingchi, 860000 China

**Keywords:** *Herpetospermum pedunculosum*, The Tibetan plateau, Altitude, Physiology, Metabolism

## Abstract

**Background:**

*Herpetospermum pedunculosum* (Ser.) Baill is annual scandent herbs. They are used in the treatment of piles, inflammation of the stomach and the intestines. It can survive the extreme environment of the Tibetan Plateau (TP). However, the underlying mechanisms of this adaptation to *H*. pedunculosum from TP remain unclear. Here, we combined physiological and metabolomics methods to analyze *H*. pedunculosum response to altitude gradient differences.

**Results:**

At high altitude, increases in the activities of Ascorbate peroxidase (APX), Glutathione reductase (GR), Dehydroascorbate reductase (DHAR), Monodehydroascorbate reductase (MDHAR), Superoxide dismutase (SOD) have been observed in leaves. Total Glutathion content, total Ascorbate content and the ASA (ascorbic acid)/docosahexaenoic acid (DHA) ration were highly elevated from low altitude to high altitude. In addition, high altitude induces decrease of the Anthocyanidin content (ANTH) and increase of abscisic acid content (ABA). The GC-MS analyses identified of 50 metabolites from leaves of *H. pedunculosum*. In addition, a metabolic network was constructed based on metabolomic datasets using a weighted correlation network analysis (WGCNA) approach. The network analysis uncovered 4 distinguished metabolic modules highly associated with I, II, III and IV respectively. Furthermore, the analysis successfully classified 50 samples into seven groups: carbohydrates, amino acids, organic acids, lipid components, polyamine, secondary metabolism and others.

**Conclusions:**

In the present study, the content of parts of amino acid components increased in samples collected at higher altitudes, and most of metabolites, including carbohydrates and organic acids were assigned to the carbon metabolic pathway comprising reductive pentose phosphate pathway, glycolysis and TCA cycle, indicating the direct relationship between adaptability and the carbon metabolic pathway and amino acids in *H. pedunculosum* response to high altitude. The results of this study laid the foundation of the molecular mechanism on *H*. pedunculosum from high altitude.

**Electronic supplementary material:**

The online version of this article (10.1186/s12864-019-5778-y) contains supplementary material, which is available to authorized users.

## Background

*Herpetospermum pedunculosum* (Ser.) Baill is key cucumber species distributing in the Tibetan plateau of China. As annual herbs, it can survive in the Tibetan Plateau (TP) extreme environment, including violent winds, low temperatures, drought and low oxygen concentration [1]. Therefore, it is an ideal material for studying biological evolution experiments. Different morphological and physiological changes in leaves were observed in alpine plants [2]. However, the responding mechanism of *H*. pedunculosum to TP has been unclear. *H*. pedunculosum is also distributed in southwest China, Nepal, and northeast India [3, 4]. In TP, their dried ripe seeds are widely used as Tibetan medicine to treat inflammatory, cholagogue and febrifuge [3]. As a drug for the treatment of piles and gastrointestinal inflammation due to its bitter taste and a cooling potency [5]. The latest research showed *H.* pedunculosum as a natural medicine for liver disease [6]. Research shows protective effect of seed oil of *Herpetospermum pedunculosum* against carbon tetrachloride-induced liver injury in rats [7]. However, the physiological and genetic adaptations responsible for its ability to cope with the harsh environment of TP remain unknown.

Increasing evidence supported that altitude gradients are a useful environmental factors to investigate ecological and evolutionary responses of plants to geographic conditions [8, 9]. Different responses have been observed in many Tibetan plants species from different altitudes [10]. For example, Hyok Chol Kim et al. found that oxygen-evolving enhancer proteins, calreticulins and S-adenosyl-L-homocysteine hydrolase can enhance the ability of plants to cope with high altitudes complex environments [11]. Wang et al. found that *Rhododendron* (Ericaceae) size and the wings of seeds become smaller at higher altitudes than at lower altitudes [12]. *P.* saundersiana has multiple strategies to adapt to the high-altitude environment of the Northwestern TP [1]. Resprouting ability and mobile carbohydrate reserve in an oak shrubland decline with increasing elevation on the eastern edge of the Qinghai-Tibetan Plateau [13]. Therefore, the potential impact of altitude change on the growth and physiology of these plants from TP may be of great importance to understand adaptive behaviors under harsh climate change. However, the underlying mechanisms of this adaptation to *H. pedunculosum* from TP remain unclear.

Metabolomics is a powerful tool to study the dynamic of small molecule metabolites. With advances in mass spectrometry, metabolomics have been used in ecological investigations and measurement the impact of factors such as climate change, disease and infection in plants [14]. In general, metabolic profiles will change in response to environmental conditions. In this review, we used metabolomics method to discuss the dynamic of metabolite accumulation of leaves of *H. pedunculosum* in response to a different altitude. However, comparative metabolomics methods have not been extensively used to study *H. pedunculosum* plant ecological adaptation. Therefore, the underlying physiological and molecular mechanisms of this adaptation of *H. pedunculosum* to the extremely Tibetan environment remain unclear.

Here, we combined physiological and metabolomics methods to analyze *H. pedunculosum* response to altitude gradient differences. Based on these results, we hypothesized that the *H. pedunculosum* plants use multiple strategies to acclimate to environmental stress from TP. The results of this study lay the foundation for *H*. pedunculosum research on the molecular mechanism of high altitude.

## Results

### Phenotypes and physiological changes

**Fig. 1:**
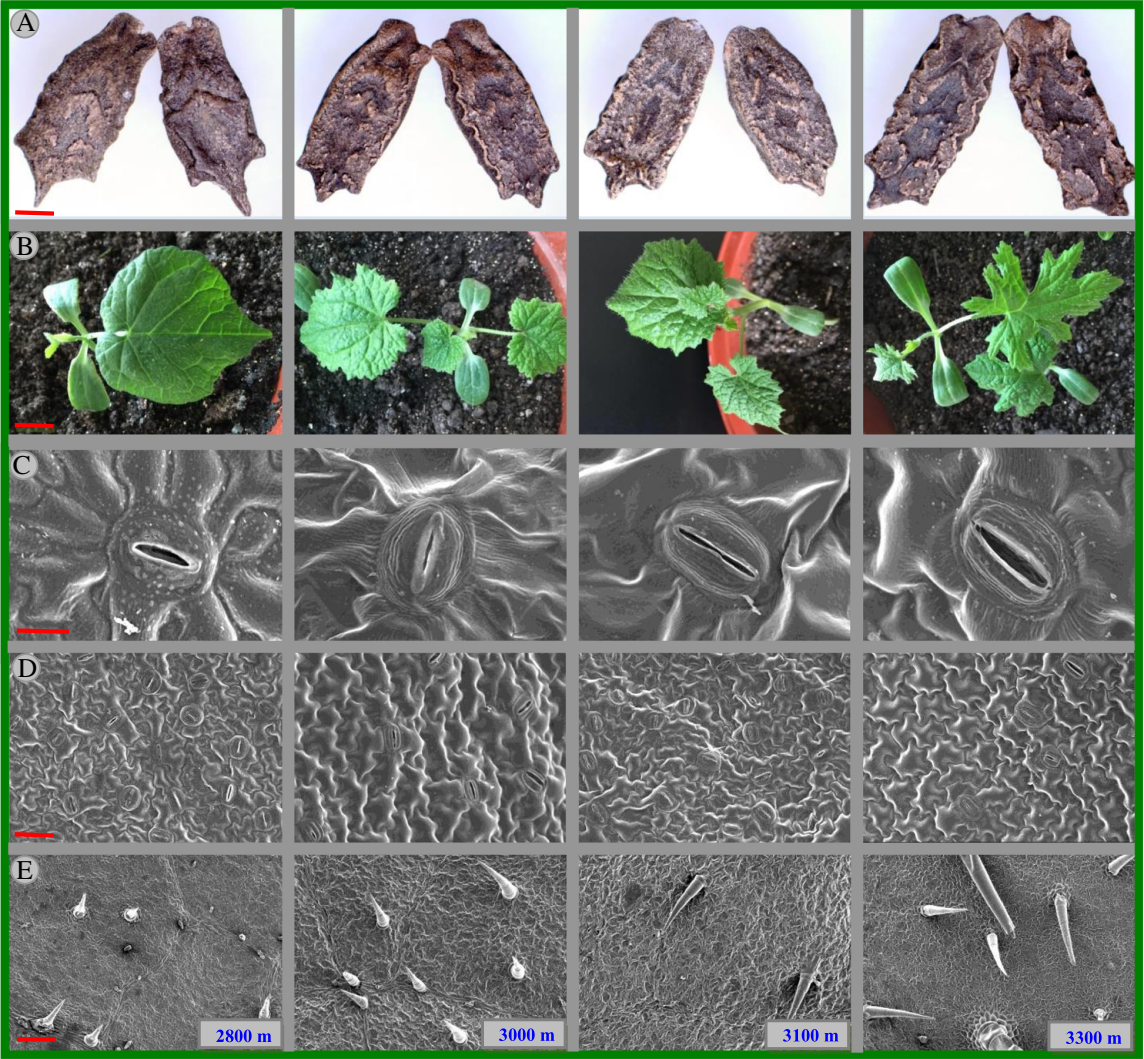
Effects of altitude gradient on the phenotype of the seeds (**a**, bar = 2 mm), leaf shape (**b**, bar = 5 cm), stomatal size (**c**, bar = 4 μm), stomatal density (**d**, bar = 10 μm) and trichomes density (**e**, bar = 100 μm) in leaf epidermis of *Herpetospermum pedunculosum* (Ser.) Baill. The plant samples were selected from different altitude (2800 m, 3000 m, 3100 m and 3300 m), respectively. The seeds (*n* = 50 for each site), the leaves size (*n* = 20 for each site), the stomatal size (*n* = 50 for each site), the stomatal density (*n* = 50 for each site) and trichomes density (*n* = 50 for each site)

The phenotypes of seeds and leaves from different samples at different altitudes were observed and compared (Fig. 1). There is poor smooth in surface of seeds from high altitude (3300 m) (Fig. 1a). The leaf shape among samples was smooth at 2800 m low altitude, while the leaf margin was jagged edge at 3000 m–3300 m high altitudes (Fig. 1b). Additionally, an increased altitude had no effect on the seed length, seed width and seed thickness (Additional file 1: Figure S1). Stomatal size and stomatal density decreased with elevated altitude (from 2800 m to 3300 m), in contrast, trichomes density increased at high altitude compared to low altitude (Fig. 1c, d, e and Additional file 1: Figure S1).

**Fig. 2:**
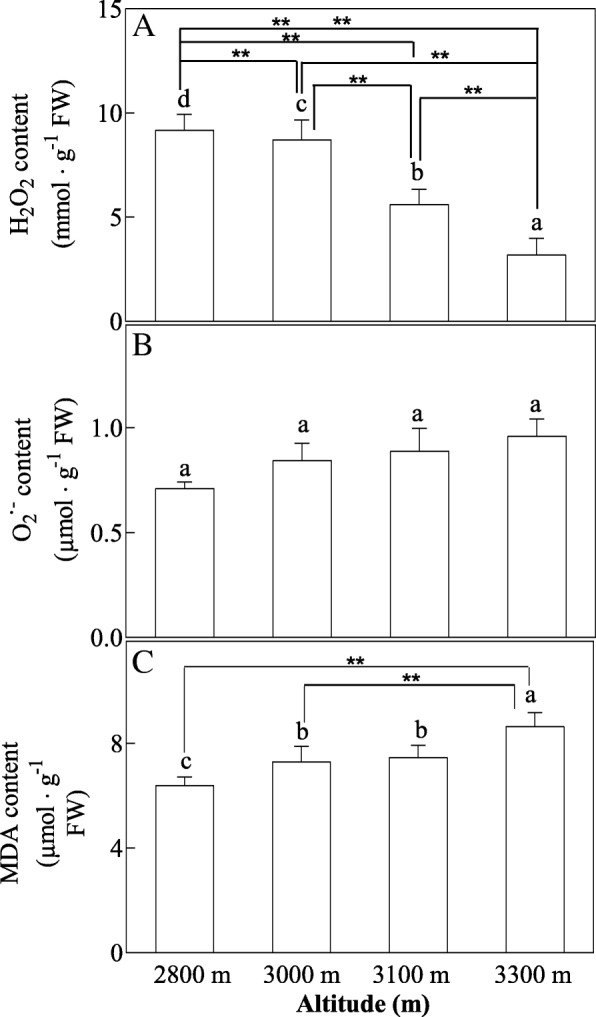
Effects of altitude gradient on the values of H_2_O_2_ content (**a**), O_2_^·-^ content (**b**) and MDA content (**c**) in leaves of *Herpetospermum pedunculosum* (Ser.) Baill. The plant samples were selected from different altitude (2800 m, 3000 m, 3100 m and 3300 m), respectively. Different letters above bars indicate statistically significant differences between different altitudes at *P* < 0.05 according to Duncan’s multiple range Test. Significant differences *P* < 0.01 are indicated two asterisks

**Fig. 3:**
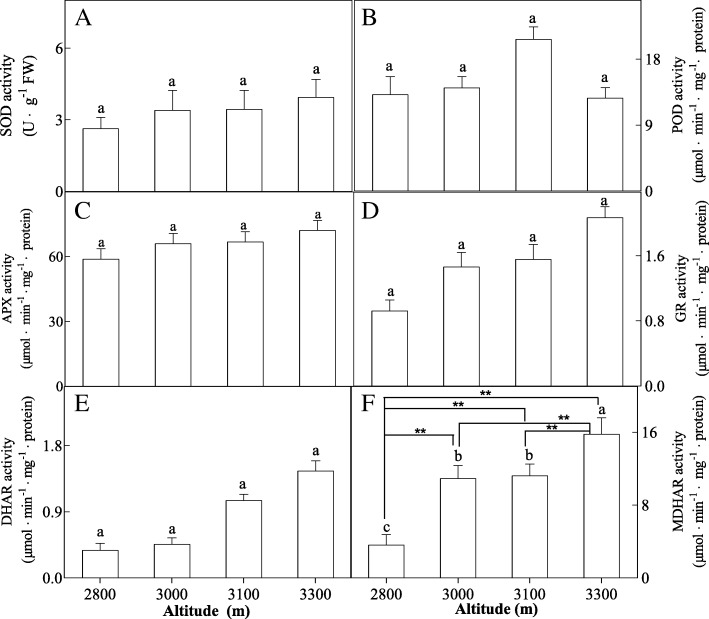
Effects of altitude gradient on the activities of SOD (**a**), POD (**b**), APX (**c**), GR (**d**), DHAR (**e**) and MAHAR (**f**) in leaves of *Herpetospermum pedunculosum* (Ser.) Baill. The plant samples were selected from different altitude (2800 m, 3000 m, 3100 m and 3300 m), respectively. Different letters above bars indicate statistically significant differences between different altitudes at *P* < 0.05 according to Duncan’s multiple range Test. Significant differences *P* < 0.01 are indicated two asterisks

**Fig. 4:**
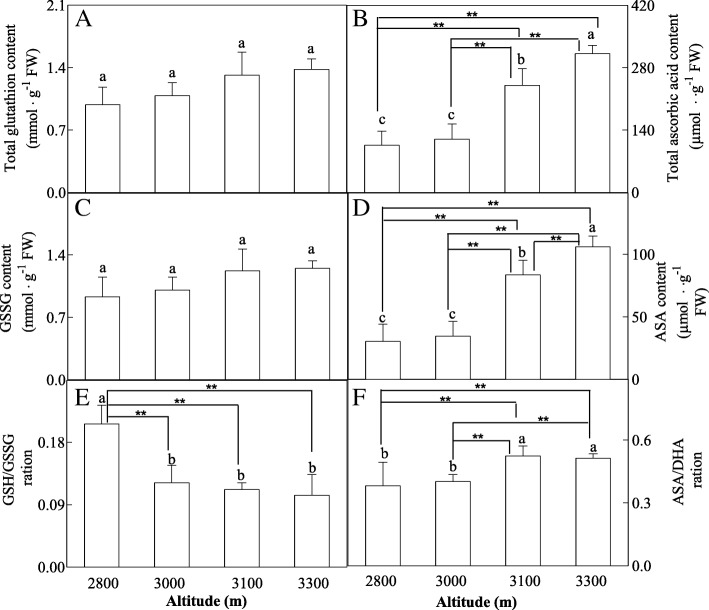
Effects of altitude gradient on the values of total glutathion content (**a**), total ascorbate content (**b**), GSSG content (**c**), ASA content (**d**), GSH/GSSG ration (**e**) and ASA/DHA ration (**f**) in leaves of *Herpetospermum pedunculosum* (Ser.) Baill. The plant samples were selected from different altitude (2800 m, 3000 m, 3100 m and 3300 m), respectively. Different letters above bars indicate statistically significant differences between different altitudes at *P* < 0.05 according to Duncan’s multiple range Test. Significant differences *P* < 0.01 are indicated two asterisks

H_2_O_2_ content was found to show decrease with increased altitude, but higher content of O_2_^−^ and MDA was found to be produced in high altitude (Fig. 2). Increased altitude induced increased activities of SOD, APX, GR, DHAR and MDHAR, but POD activity was gradually increased with increased altitude, and reached to the maximum value at 3100 m altitude, and then decreased at 3300 m altitude (Fig. 3). Total Glutathion content, total ascorbate content, GSSG content and ASA content were increased with increased altitude (Fig. 4). However, GSH/GSSG ration decreased with increased altitude (Fig. 4).

**Fig. 5:**
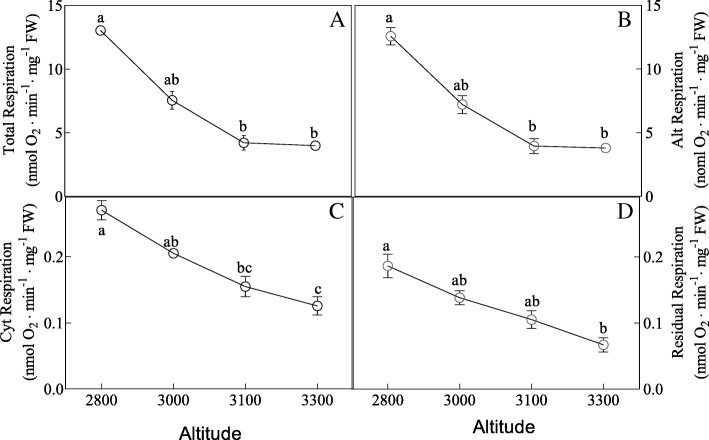
Effects of altitude gradient on the values of Total respiration rate (**a**), Alt respiration rate (**b**), Cyt respiration rate (**c**) and Residual respiration rate (**d**) in leaves of *Herpetospermum pedunculosum* (Ser.) Baill. The plant samples were selected from different altitude (2800 m, 3000 m, 3100 m and 3300 m), respectively. Different letters above bars indicate statistically significant differences between different altitudes at *P* < 0.05 according to Duncan’s multiple range Test

**Fig. 6:**
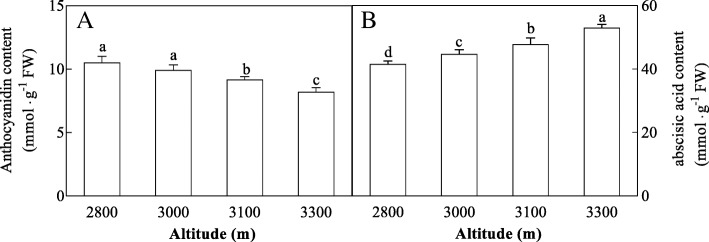
Effects of altitude gradient onanthocyanidin content (ANTH, **a**) and abscisic acid content (ABA, **b**) in leaves of *Herpetospermum pedunculosum* (Ser.) Baill. The plant samples were selected from different altitude (2800 m, 3000 m, 3100 m and 3300 m), respectively. Different letters above bars indicate statistically significant differences between different altitudes at *P* < 0.05 according to Duncan’s multiple range Test

In addition, high altitude was found to cause a decrease in the total respiratory rate, alt respiration rate, cyt respiration rate and residual respiration rate (Fig. 5). To examine the effects of high altitude on photosynthesis (*P*n, *G*s, *C*i and *T*r) and chlorophyll fluorescence of *H. pedunculosum*. we measured the dynamic *P*n, *G*s, *C*i, *T*r and the ratio of variable and maximal fluorescence (Fv/Fm) (Additional file 2: Table S1). *P*n and *G*s showed an increase at moderate altitude (3000 m and 3100 m) compared with the samples from low and high altitude (2800 m and 3300 m). In addition, *C*i was decreased with increased altitude. *T*r and *F*v/*F*m was not significantly changed between different altitudes. Additionally, the anthocyanidin content (ANTH) decreased and abscisic acid content (ABA) increased with elevated altitude (Fig. 6).

### Orthogonal projection to latent structures discriminant analysis (OPLS-DA) and heatmap construction

Analysis of total metabolite content by gas chromatography-mass spectrometry(GC-MS) for samples collected at different altitudes. After Orthogonal projection to latent structures discriminant analysis (OPLS-DA) was conducted to characterize changes in metabolite concentrations according to both identified and unidentified metabolites. OPLS-DA modeling could separate all samples into four distinct groups (Additional file 3: Figure S2) based on different altitudes. For instance, samples from low altitude (2800 m) could be separated from other high altitudes (3000 m and 3100 m). Therefore, this analysis revealed a closer clustering among biological replicates from four altitudes.

**Fig. 7:**
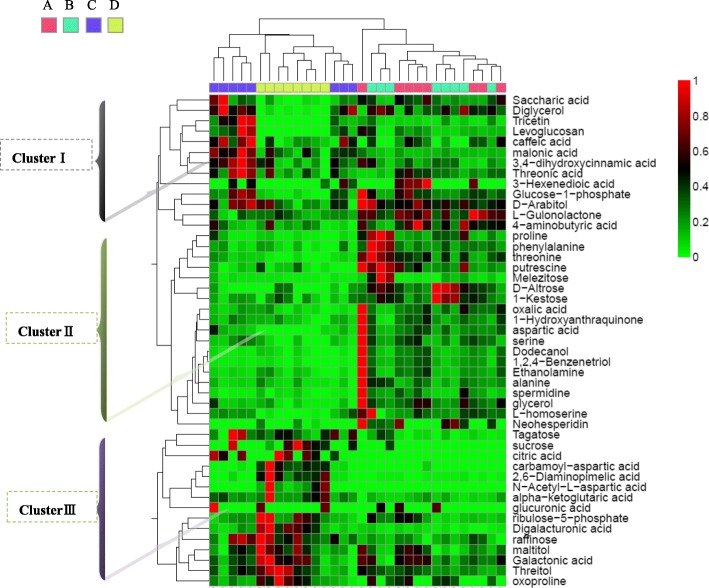
Heat map and cluster tree of identified metabolite concentrations in extracts from leaves of *Herpetospermum pedunculosum* (Ser.) Baill. Data of the content value of each compound were normalized to complete linkage hierarchical clustering. Each altitude is visualized in a single colume and each metabolite is represented by a single row: **a**, samples collected in 2800 m; **b**, samples collected in 3000 m; **c**, samples collected in 3100 m; **d**, samples collected in 3300 m, as shown at the top of the figure. Red indicates high abundance, whereas low relative compounds are blue (color key scale on the right of heat map)

To gain more insights into the modulation of metabolites in leaves from different altitudes, the metabolic data were visualized using a clustered heat map that charted the changes in metabolite concentrations between different altitudes (Fig. 7). The data for each metabolite were normalized against the lowest measured concentration within the same altitude, unlike Additional file 4: Table S2. In Additional file 4: Table S2, the values for metabolites in the heat map are used as controls at the 2800 m altitude. In Cluster I, the concentration of metabolites from four altitudes changed little. Interestingly, some metabolites from high altitude (3000 m and 3100 m) exhibited higher concentration in Cluster II. In contrast, some metabolites from high altitude showed lower levels in Cluster III. These same ways changed in some metabolites concentrations at 3000 m and 3100 m altitude, indicated that a group of metabolites is involved in high altitude changes.

### Identification of the altitude-responsive metabolites in leaves

To further analyze the metabolic response between different altitudes, the relative contents of these different identified metabolites in leaves of *H. pedunculosum*. Were normalized to the contents found at 2800 m (Additional file 4: Table S2 and Additional file 5: Table S3). Here, 50 metabolites were ambiguously annotated by comparing the putative metabolite mass spectra. They were divided into seven groups: carbohydrates, amino acids, organic acids, lipid components, polyamine, secondary metabolism and others (Additional file 4: Table S2).

**Fig. 8:**
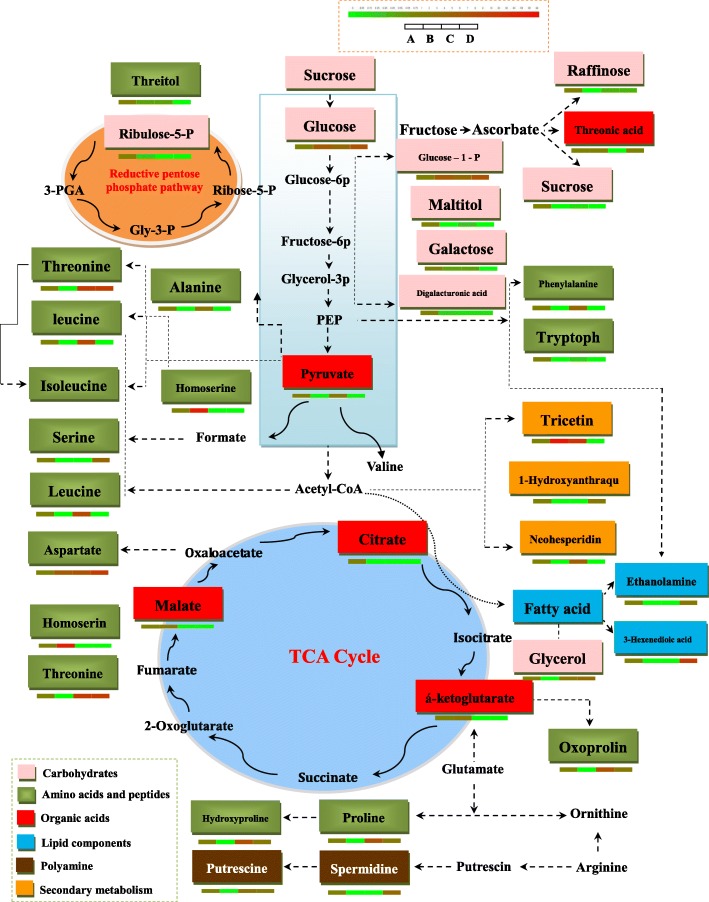
Primary metabolism pathways of measured metabolites in leaves of *Herpetospermum pedunculosum* (Ser.) Baill.Represented pathways are simplified versions of the tricarboxylic acid (TCA) cycle, carbohydrates, amino acid and organic acids. Each box represents one metabolite. Carbohydrates, amino acid, organic acids, lipid components, polyamine and secondary metabolism are displayed in pink, green, red, blue, brown and orange. Each column represent different altitudes (from left to right: **a**, 2800 m, **b**, 3000 m, **c**, 3100 m, **d**, 3300 m) which is shown in the upper left corner. Average metabolite intensity is color coded according the scale in the upper right corner

Most of the identified metabolites (50, 70%) exhibited increases in abundance in high altitude (3000 m, 3100 m and 3300 m) (Additional file 4: Table S2 and Additional file 6: Figure S3). However, 15 metabolites showed decreases in abundance. The most pronounced increases in abundance were observed for secondary metabolism (Tricetin, 80 ×). In addition, carbohydrates and amino acids also showed high abundance. Metabolites that were accumulated in levels were tricetin (whose abundance increased (83.85 ×), L-homoserine (61.46 ×), levoglucosan (40.09 ×), threonine (18.68 ×) and diglycerol (14.32 ×). By contrast, the most pronounced decreases in abundance were observed for sucrose (0.10 ×), threitol (0.62 ×), raffinose (0.42 ×), galactonic acid (0.63 ×), digalacturonic acid (0.19 ×), ribulose-5-phosphate (0.31 ×), maltitol (0.39 ×), tagatose (0.48 ×), 2,6-diaminopimelic acid (0.05 ×), oxoproline (0.28 ×), carbamoyl-aspartic acid (0.05 ×), 5-hydroxytryptophan (0.30 ×), alpha-ketoglutaric acid (0.28 ×) (Additional file 4: Table S2 and Fig. 8). In addition, 5 metabolites, including saccharic acid, glucose-1-phosphate, levoglucosan, digalacturonic acid, aspartic acid showed changes in levels at all altitudes (3000 m, 3100 m and 3300 m) (Additional file 4: Table S2 and Additional file 3: Figure S2). Then we further characterized the changes in different metabolites based on pairwise comparisons of peak area (Additional file 7: Figure S4). Compared to 2800 m altitude, there are significant changes in the concentrations of 34 (among identified 103 compounds) and 33 (among 113) metabolites from 3100 m and 3300 m altitude, respectively. The smallest change occurred between A and B altitude, there are only 21 (among 134) metabolites changed (Additional file 7: Figure S4).

### Pathway mapping and the metabolite-to-metabolite network visualization

**Fig. 9:**
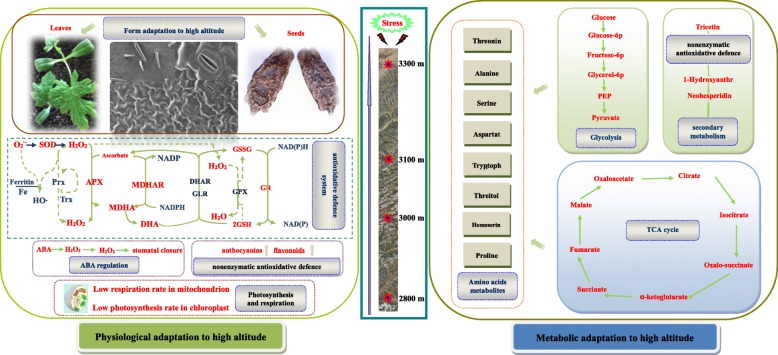
Schematic presentation of systematic tolerance mechanisms in *Herpetospermum pedunculosum* (Ser.) Baill to high altitude. The identified proteins were integrated into subcellular locations and pathways. Abbreviations: ABA, abscisic acid; APX, ascorbate peroxidase; DHA, dehydroascrobate; DHAR, DHA reductase; Fructose-6p, fructose-6-phosphate; Glucose-6p, glucose-6-phosphate; Glycerol-6p, Glycerol-6-phosphate; GPX, glutathione peroxidase; GR, glutathione reductase; GSH, reduced glutathione; GSSG, oxidized glutathione; GST, glutathione S-transferase; MDHA, monodehydroascorbate; MDHAR, MDHA reductase; NADP, nicotinamide adenine dinucleotide phosphate; NADPH, triphosphopyridine nucleotide; PEP, phosphoenolpyruvate; Prx, peroxiredoxin; TCA cycle, tricarboxylic acid cycle; Trx, thioredoxin

To obtain a detailed and clear overview on the abundance of differences and/or similarities of the identified metabolites in leaves from different altitudes, a simplified version of primary metabolism pathways was constructed to compare the behavior of the main metabolites between different altitudes (Fig. 8 and 9). Figures 8 and 9 showed the metabolic pathways leading to the most primary metabolites involved in the TCA cycle, reductive pentose phosphate pathway and glycolysis pathway.

### WGCNA correlation network construction

Analysis of total metabolite content by gas chromatography-mass spectrometry for samples collected at different altitudes. After manual inspection, recalculation of multiple peaks and exclusion of peaks originating from internal standards, 249 putative metabolites was identified. All of the metabolites identified in leaves of *H. pedunculosum*. Are shown in Additional file 8: Table S4.

**Fig. 10:**
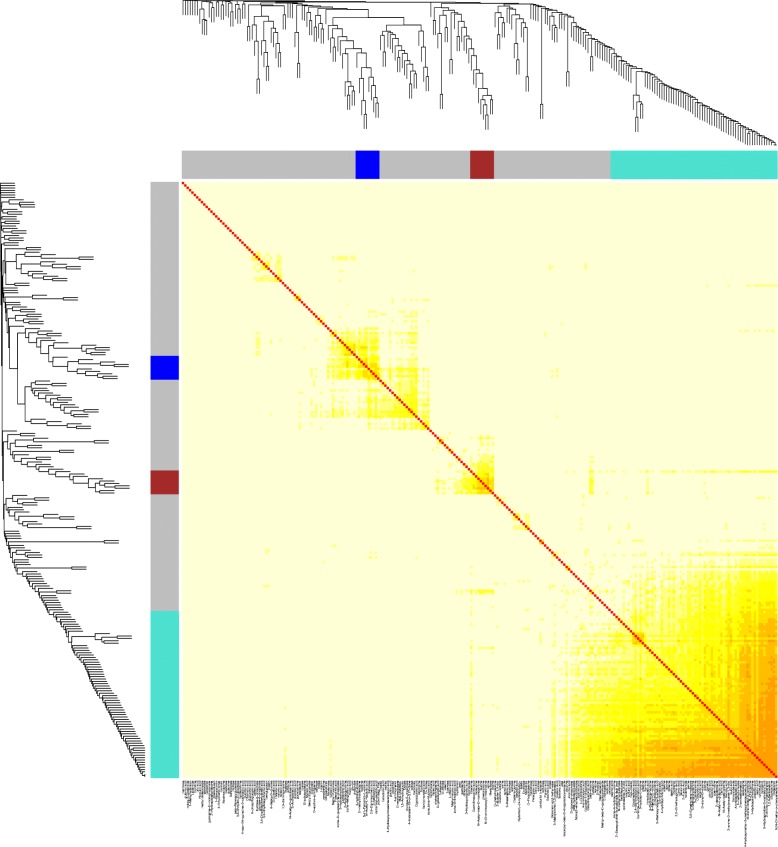
WGCNA of meabolomic profiles of *Herpetospermum caudigerum* Wall. leaves from different altitude gradient in the Tibetan Plateau

A weighted correlation network analysis (WGCNA) approach was used to identify metabolic modules related to a different altitude by the GC-MS data. Thus, networks were constructed based on all identified metabolites. Accordingly, all the correlated metabolites were grouped into 4 distinct metabolic modules (I, II, III and IV) (Fig. 10). Among them, Module I (D-Glyceric acid is the most abundant), II (Proline is the most abundant), III (Melezitose is the most abundant) and IV (Neohesperidin is the most abundant) have 15, 43, 27 and 87 metabolites respectively (Fig. 10). Every module consisted of most of carbohydrates, amino acids and organic acids.

## Discussion

To understand the mechanical properties in response to high altitude, the stomatal size and stomatal density in leaf epidermis was measured. The pollutant ozone and pathogenic microbes can result in stomatal closure and thereby prevent damaging entry to the leave [15–17]. As shown in Fig. 1 and Additional file 2: Table S1, at high altitude, stomatal size and stomatal density showed a decrease tendency. This decrease might help plants to reduce damage from high altitude. Indeed, previous reports indicated that the stomatal aperture in *P.* saundersiana and *A. alpina* decreased at high altitude [1, 18]. However, Kjær et al. indicated that stress treatment of *A. annua* plants had a minor promoting effect on more numerous glandular trichomes [19]. In contrast, the study of Adebooye et al. showed that it is likely that salinity has much of an effect on glandular trichome density [20]. Thus, it is need to study further that the increase in number of glandular trichomes might be related to the fine regulation of the tolerance in *H. pedunculosum* at high altitude.

Overproduction of ROS such as H_2_O_2_ and O_2_^·-^ can result in subsequent oxidative stress, which can lead to reduction in photosynthetic CO_2_, pigment loss and decreased protein [21]. In Fig. 2, the contents of H_2_O_2_ and O_2_^·-^ varied slightly with elevated altitud. These observations confirmed that *H. pedunculosum* plants at high altitude suffer less oxidative stress, which is potentially linked to plant adaptation and tolerance to biotic and abiotic stressed in Tibet. However, there results were not consistent with the finding for an indicator of membrane lipid peroxidation (MDA). Additionally, previous research found that increased altitude resulted in marked increases in H_2_O_2_ and O_2_^·-^ in plant *Lamiophlomis rotata* and *Populus kangdingensis* [1, 22]. These inconsistencies are lack some reasonable explanation. Therefore, further studies are needed to investigate this mechanism. Or *H. pedunculosum* at high altitude can take advantage of various antioxidative defense systems to defense oxidative stress.

In Fig. 3, increases in the activities of APX, GR, DHAR, MDHAR, SOD have been observed in leaves with increased altitude. And in Fig. 4 the ascorbate and glutathione cycle have been also observed. Total glutathion content, total ascorbate content and the ASA/DHA ration were highly elevated from low altitude to high altitude. This suggested that the importance of the regulation at different antioxidant mechanism to cope with the harsh environment at high altitudes. High level of glutathione and ascorbate enhances plant stress by modulating the antioxidant [23, 24].

The energy required for biosynthesis is solely generated from respiration in plants [25]. Thus, the ability to maintain respiration metabolism can represent a positive trait for plant survival under stress environment [26]. Interestingly, as shown in Fig. 5 leaf respiration rate including total respiration, alt respiration, cyt respiration and resdual respiration in leaves of *H. pedunculosum* at high altitude all showed decreased tendency with elevated altitude. As shown in Fig. 9 results may be attributed to reduction of energy consumption to maintain normal growth under environmental stress, and also reflecting the low energetic demands of adapting of *H. pedunculosum* to high altitude. Our conclusion is consist with the study of Habibi et al. [27]. Therefore, from previous studies, there is no clear expectation of the respiratory rate response in relation to the variation in whole-plant tolerance. The values of *P*n and *G*s were lower at low altitude (2800 m), then increased at moderate altitude (3000 m and 3100 m), and last decreased at high altitude (3300 m). Parts of results are in agreement with previous reports alpine *Phrynocephalus erythrurus* [28] and *P.* saundersiana [1]. At the same time, internal CO_2_ concentration (*Ci*) exhibited low levels with increased altitude, which would affect photosynthetic activity. Indeed, our metabolomic analysis showed the content of ribulose-5-phosphate was decreased. As an important component, maintenance of ribulose-5-phosphate was linked to photoprotection under stress [29]. This photoprotection was possibly due to nor other environmental factors rather stomata or altitude factors.

Extensive studies have also revealed that plants can adapt to different environmental stressed by ABA regulation [30–33]. There is a close linear correlation between the ABA content of the leaves and their conductance (stomatal resistance) [34]. In this study, there were increases of ABA content and decrease of stomatal size in leaves with decreased altitude. Our conclusion is consist with the study of Bano et al. [35] Indeed, in plants, first ABA is produced from the roots under stems, then translocated to the leaves, the osmotic potential of stomatal guard cells was changed, leading to close stomata [36].

In the study, we firstly reported that metabolomic involvement in response to altitude in *H. pedunculosum*. Generally, primary metabolites are directly involved in plant growth and development. Amino acids also play a role in the defense mechanisms and stress responses of plants [37–39]. The changes in concentration of carbohydrates, amino acids as well as organic acids and so on in leaves of *H. pedunculosum*, which were indicated in Additional file 4: Table S2 and Fig. 8. In the present study, the content of parts of amino acid components increased in samples collected at higher altitudes, suggesting a role for osmolyte, regulation of ion transport in the plant’s adaptation to high altitude environmental stress. As previously reported, plants subjected to under various stresses showed accumulation of some amino acids. In plants, amino acids play several critical roles including providing the building blocks of proteins and shuttling organic nitrogen [40]. Additionally, amino acids also affect gene expression, activity of some enzymes and redox-homeostasis [41]. These roles played by amino acids suggested that high levels of amino acids in plants could enhance high altitude tolerance of *H. pedunculosum*. However, further studies will be needed to clarify the functions of these amino acids in high altitude tolerance. By contrast, the levels of some amino acids such as oxoproline, carbamoyl-aspartic acid and 5-hydroxytryptophan were reduced in plant samples collected at high altitudes. The reductions in these amino acids may contribute to the production of new essential stress proteins or repair damaged or misfolded proteins to facilitate *H. pedunculosum* for acclimation high altitude. Generally, for central role in nitrogen metabolism, biosynthesis, degradation, and transport of amino acid are tightly regulated to meet demand to nitrogen and carbon availability [40]. Or these differentiated changes of amino acids may contribute directly to the improved tolerance of *H. pedunculosum* from high altitude, as this different accumulation of these amino acids under high altitude is energetically more efficient when the biosynthesis and degradation of amino acids occur in leaves simultaneously. However, the metabolism, transport, and stress signal integration of these amino acids can influence each other in an unpredictable pattern up to now.

The dose-dependent elevation in amino acid levels suggest that it is more accumulated than other metabolites, and their accumulation is a more sensitive response to altitude changes. Among all identified metabolites from different altitudes, four secondary metabolites also showed increased level. An extreme metabolite was varied in this study. For instance, tricetin exhibited the largest fold-change (83.85 ×). In plants, the flavones tricetin is relatively rare and was only reported to occur in the myrtaceae pollen [42]. However, some reviews indicated that tricetin is more widespread than was known so far [43, 44]. For instance, in wheat, rice and barley tricetin has also been detected. The tolerance capability of tricetin occurrence in plants may be ascribed to its potential genes play an important role in plant-environmental biotic/abiotic stress [45–47]. Although no direct evidence showed that tricetin could serve as a secondary metabolites induced by environmental stress, tricetin methylation may play an important role under abiotic stresses [48].

The metabolic profile analyses demonstrated that the high altitude of resulted in substantial Among them, tricarboxylic acid (TCA) cycle intermediates supporting diverse functions including defense, developmental stages and tissue types in plants [49, 50]. Interestingly, most of metabolites including carbohydrates and organic acids were assigned to the carbon metabolic pathway comprising reductive pentose phosphate pathway, glycolysis and TCA cycle, indicating the direct relationship between adaptability and the carbon metabolic pathway in *H. pedunculosum* response to high altitude. Physiologically, some of these metabolites can play a role act as osmolytes in cytoplasm under extreme environment in Tibet, resulting in an increase of osmotic potential of the cytoplasm in leaf cells to maintain appropriate cell status, while the accumulation of organic acids may contribute to keeping ionic balance [51].

Although much is known about the regulation mechanism of biosynthesis pathways by metabolites themselves, it should be pointed out that only parts of metabolites were measured in this study. Therefore, the regulation mechanisms at the transcriptional, post-transcriptional, and protein levels to more metabolites need to be identified. In addition, it is somewhat regrettable that we can′t present the detailed data including temperature, rainfall and soil because of the special geographical environment. Accordingly, the relationship between environment and adaptability of *H. pedunculosum* to Tibetan environment is completely clear. Therefore, we should synthetically consider these factors in the next work.

## Conclusions

To date, *H. pedunculosum* can survive harsh condition in the Tibetan Plateau, however the adaptation mechanism to adverse environment remains unknown. To gain a comprehensive physiological and biochemical and metabolomic understanding of *H. pedunculosum*, we compared physiological and comparative metabolomic traits of different altitudes *H. pedunculosum*. Differences in seed and leaf phenotypes at different altitudes. H_2_O_2_, O_2_^−^, MDA, SOD, APX, GR, DHAR, MDHAR, GSSG, AsA content was found to show increase with increased altitude. In addition, high altitude was found to cause a decrease in the total respiratory rate, at respiration rate, cyt respiration rate and residual respiration rate.

Most of the metabolic species identified by metabolomics are carbohydrates, amino acids and organic acids. To obtain a detailed and clear overview on the abundance of differences, we have drawn metabolic pathways Figs. 8 and 9. In the present study, the content of parts of amino acid components increased in samples collected at higher altitudes, suggesting a role for osmolyte, regulation of ion transport in the plant’s adaptation to high altitude environmental stress. The most of metabolites, including carbohydrates and organic acids were assigned to the carbon metabolic pathway comprising reductive pentose phosphate pathway, glycolysis and TCA cycle, indicating the direct relationship between adaptability and the carbon metabolic pathway in *H. pedunculosum* response to high altitude.

## Methods

### Experimental samples collection

The experiments were carried out at the Harbin Northeast Forestry University Genetic laboratory. The leaf and seeds of the *H. pedunculosum* are collected in August 2016 Gangdese Mountains (29°64′N, 94°37′E) near Bayi District, in central Tibet Autonomous Region, China.

### Seed and leaf morphology

The surface texture observation of 30 individual seed at each altitude randomly collected by digital camera (Olympus SZX7, Olympus Corporation, Japan). And leaves in seedlings were also investigated by photograph. Three exomorphic parameters including seed length, seed width, seed thickness were detected and thousand of seed weight was also calculated (Fig. 1 and Additional file 2: Table S1).

For scanning electron microscopy (SEM), the randomly selected leaves were from plants at the same and different altitudes, washed double-distilled water and were fixed in formaldehyde. The sample was dehydrated with ethanol (70, 85, 95 and 100%) and then sprayed with gold. Then, observations and photographs were obtained using a SEM (JSM-5310LV, Japan). The stomatal shape and size including stomatal length, stomatal density and trichomes density was calculated with Image J 1.4.7 software (Image J. Bethesda, Maryland, USA).

### Measurements of H_2_O_2_, O_2_^• −^ and MDA

Hydrogen peroxide (H_2_O_2_) was detected spectrophotometrically according to PV Sergiev et al. [52]. 1.5 g fresh samples were ground into powder by liquid nitrogen and added into 4 ml 1% TCA ice water bath for 5 min. After centrifugation at 6, 000×*g* for 10 min the supernatant was aspirated. 0.5 ml PBS and 1 ml potassium iodide were added to the 0.5 ml extracted solution for dark reaction for 5 min. The absorbance of the mixture was measured at 390 mm and H_2_O_2_ concentration was obtained according to the standard curve of hydrogen peroxide.

0.5 g fresh leaves are ground into powder in liquid nitrogen and added into PBS buffer, after centrifugation at 6, 000×*g* for 10 min the supernatant was aspirated. 0.5 ml phosphate buffer solution (65 mM) and 0.5 ml hydroxylamine hydrochloride (10 mM) were added to 1 ml extracted solution and keep 25 °C for 0.5 h. Add developer 1 ml α-naphthylamine to the mixture and dark reaction for 15 min. The absorbance of the mixture was measured at 530 mm and O_2_^• −^ generation rate was obtained according to the standard curve of sodium nitrite.

MDA content was estimated by the method of Wang et al. [53]. 1.5 g fresh samples were ground into powder by liquid nitrogen and added into 4 ml 1% TCA ice water bath for 5 min. After centrifugation at 6, 000×*g* for 10 min the supernatant was aspirated. Add 2 ml TBA (0.5%) to 1 ml extracted solution and then boil in water for 15 min and centrifuged at 12, 000×*g* for 5 min. Absorbance was measured at 450, 532 and 600 nm. Statistical analysis of measurement results by SPSS software.

### Measurements of enzymatic activities

Superoxide dismutase (SOD, EC1.15.1.1) activity was determined according to the method of Beauchamp and Fridovich [54]. Peroxidase (POD, EC 1.11.1.7) activity was measured by the method of Bisht et al. [55]. 0.4 g fresh samples were ground into powder by liquid nitrogen and added into 4 ml PBS ice water bath for 5 min. After centrifugation at 6, 000×*g* for 10 min the supernatant was aspirated. The extracted solution of 60 μl was added with 0.2 ml MET, 0.2 ml EDTA, 0.2 ml NBT and 0.3 ml riboflavin reaction for 15 min. The absorbance of the mixture was measured at 560 mm. Ascorbate peroxidase (APX, EC1.11.1.11) activity was measured by the method of Y, Nakano and K Asada [56]. The extracted solution of 50 μl was added with 200 μl ASA and 500 μl H_2_O_2_. The absorbance of the mixture was measured at 290 mm. Glutathione reductase (GR, EC 1.6.4.2) was measured following the method of Carlberg [57]. The extracted solution of 300 μl was added with 1550 μl PBS, 140 μl NADPH and 260 μl GSSG. After the absorbance of the mixture were measured at 290 mm. Dehydroascorbate reductase (DHAR, EC 1.8.5.1) was measured following the method of Dalton [58]. Monodehydroascorbate reductase (MDHAR, EC 1.6.5.4) was measured following the method of Hossain [59]. Statistical analysis of measurement results by SPSS software.

### Measurements of non-enzymatic antioxidants

The content of total ascorbic acid (AsA + DHA) was determined following the method of Kampfenkel et al. [60]. The absorbance was measured at 525 nm and ASA concentration was obtained according to the standard curve of ASA. Accordingly, total ascorbate (AsA + DHA) was determined. DHA content was calculated from the difference between total ascorbate and AsA.

Total glutathione (the oxidized, GSH + the oxidized, GSSG) were determined following the method of Edwards et al. [61]. The absorbance was measured at 412 nm and GSH concentration was obtained according to the standard curve of GSH. Statistical analysis of measurement results by SPSS software.

### Measurements of gas exchange parameters and respiratory measurements

At 9:30 am Li-Cor 6400 portable photosynthesis measuring systems (LI-Cor Inc., Lincoln, NE) was used to measure the gas exchange parameters (stomatal conductance (*Gs*), maximum efficiency of PSII (*Fv/Fm*), Net photosynthetic rate (*Pn*) and intercellular CO_2_ (*Ci*)) of leaves at 25 °C, photon f1ux density (PFD) 900 μmol m^− 2^ s^− 1^ [53].

The Leaf respiration rate was determined following the method of Tomaz et al. [62]. 1 cm^2^ leaf segments were in the dark for 30 min and then submerged in 2 ml buffer (10 mM MES, 2 mM CaCl_2_ and 10 mM HEPES). After the total respiration rate, SHAM-resistant respiration (Cyt pathway) rate and KCN-resistant respiration (alternative pathway) were measured.

### Hormone and pigment determination

0.5 g frozen samples were ground into powder by liquid nitrogen and added into buffer (80% methanol, 100 μg butylated hydroxytoluene) ice water bath for 4 h and the supernatants were obtained by centrifugation for 15 min (3500 rpm/min, 4 °C). The supernatant was separated by C18 columns to obtain the hormone. Endogenous MeJA level was determined following the method of Bai et al. [63]. The total monomeric anthocyanin concentration is mensurated with a validation pH differential method discribed.

### GC-MS analysis of metabolites

The metabolites from each sample were extracted by the methanol/chloroform method. 60 mg leaves were ground into powder by liquid nitrogen and added into extract buffer. Then the homogenate was vortexed and sonicated for 30 min, twice and centrifuged (4 °C, 14000 rpm, 10 min). 700 μL supernatant was air-dried, added by 80 μL pyridine amine hydrochloride (15 mg/ml) and vortexed for 1.5 h at 37 °C. Then the mixture was added buffer (20 μL hexane, 80 μL *N*,*O*-bis (trimethylsilyl) -trifluoroaceta by mid (BSTFA) and 1% TMCS), GS-MS analysis for metabolitic analysis.

The metabolites were analyzed by Agilent 7890A-5975C GC-MS system (Agilent Technologies, Inc. Santa Clara, CA) with non-polar DB-5 capillary column (30 m × 250 μm I.D. J&W Scientific, Folsom, CA). The mass spectrometer was set to scan a mass range of 50–600 *m*/*z* at 20 scans/s using a 70 eV electron beam [64].

All unprocessed data from GC-MS were analyzed by C_HROMO_ TOF 4.34 (LECO Corp.) software package. Metabolites (Variable importance in the projection, VIP > 1.0 and *p* < 0.05) were identified based on available reference standards in our lab and the NIST 11 mass spectra library (National Institute of Standards and Technology, Gaithersburg, MD) and the Fiehn library linked to C_HROMO_ TOF 4.34 software. Identification required a minimum forward match percentage of 70% or higher [64].

The SIMCA-P + 14.0 software package(Umetrics AB, Umeå, Sweden) analyzes the data set generated from GC-MS. Orthogonal projections to latent structure discriminant analysis (OPLS-DA) were carried out to visualize the metabolic alterations among experimental groups. Heat map was used to visualize metabolite response. Metabolites (fold ≥2, p < 0.05) annotated to metabolic pathway using the KEGG (Kyoto Encyclopedia of Genes and Genomes, www.genome.jp/kegg) database to identify metabolic pathways influenced by altitude. All metabolic correlation networks were carried out by the standard procedure of WGCNA [65].

## Additional files

Additional file 1: **Figure S1.** Effects of altitude gradient on the Seed length (A), Stomatal length (B), Seed width (C), Stomatal density (D), Seed thickness (E) and Trichomes density (D) of *Herpetospermum pedunculosum* (Ser.) Baill. (DOC 1830 kb)

Additional file 2: **Table S1.** Effects of altitude gradient on net photosynthetic rate (*P*n), stomatal conductance (*G*s), intercellular CO_2_ concentration (*C*i), transpiration (*T*r) and the maximum efficiency of PSII (*F*v/*F*m) in leaves of *Herpetospermum pedunculosum* (Ser.) Baill. (DOCX 1630 kb)

Additional file 3: **Figure S2.** Orthogonal projections to latent structures discriminant analysis (OPLS-DA) score scatter plot derived from the GC-MS data set for leaves of *Herpetospermum pedunculosum* (Ser.) Baill. (DOCX 98 kb)

Additional file 4: **Table S2.** The relative contents of the identified metabolites in leaves of *Herpetospermum pedunculosum* (Ser.) Baill. (DOC 97 kb)

Additional file 5: **Table S3.** on metabolites identified in Herpetospermum caudigerum Wall., showing their molecular masses, KEGG compound codes, suggested metabolic functions, and the corresponding KEGG pathway codes. (XLS 31 kb)

Additional file 6: **Figure S3.** Changes in metabolite concentrations in leaves of *Herpetospermum caudigerum* Wall. at four altitudes including 2800 m, 3000 m, 3100 m and 3300 m (A, samples collected in 2800 m; B, samples collected in 3000 m; C, samples collected in 3100 m; D, samples collected in 3300 m). B/A: The ratio of the B concentration to the A concentration is shown by red colored bars. (DOC 1120 kb)

Additional file 7: **Figure S4.** Classification of metabolites according to different altitudes. (DOCX 100 kb)

Additional file 8: **Table S4–1.** All of the metabolites identified in leaves of *Herpetospermum pedunculosum* (Ser.) Baill. **Table S4–2.** The difference of metabolites in *Herpetospermum pedunculosum* (Ser.) Baill leaves between 3000 m and 2800 m altitude. **Table S4–3.** The difference of metabolites in *Herpetospermum pedunculosum* (Ser.) Baill leaves between 3100 m and 2800 m altitude. **Table S4–3.** The difference of metabolites in *Herpetospermum pedunculosum* (Ser.) Baill leaves between 3300 m and 2800 m altitude. (XLSX 336 kb)

## Abbreviations

ABA: Abscisic acid
ANTH: Anthocyanidin content
APX: Ascorbate peroxidase
ASA: Ascorbic acid
*Ci*: Intercellular CO_2_
DHAR: Dehydroascorbate reductase
*Fv/Fm*: Efficiency of PSII
GR: Glutathione reductase
*Gs*: Stomatal conductance
GSH: Glutathione
GSSG: Oxidized glutathione
H_2_O_2_: Hydrogen peroxide
MDHAR: Monodehydroascorbate reductase
*Pn*: Photosynthetic rate
POD: Peroxidase
ROS: Reactive oxygen species
SEM: Scanning electron microscopy
Ser: Herpetospermum pedunculosum
SOD: Superoxide dismutase
TBA: Thiobarbituric acid
TCA: Tricarboxylic acid
TP: Tibetan Plateau
*T*r: Transpiration rate
UV: Strong ultraviolet
WGCNA: Weighted correlation network analysis
