# Supplementary material for: The adaptation strategies of Herpetospermum pedunculosum (Ser.) Baill at altitude gradient of the Tibetan plateau by physiological and metabolomic methods
Source: BMC Genomics. 2019 Jun 3;20:451. doi: 10.1186/s12864-019-5778-y (PMC6547600; doi:10.1186/s12864-019-5778-y)
Supplement: Supplementary file 1 — Figure S1. Effects of altitude gradient on the Seed length (A), Stomatal length (B), Seed width (C), Stomatal density (D), Seed thickness (E) and Trichomes density (D) of Herpetospermum pedunculosum (Ser.) Baill. (DOC 1830 kb) [file 12864_2019_5778_MOESM1_ESM.doc]

**Figure S1** Effects of altitude gradient on the Seed length (A), Stomatal length (B), Seed width (C), Stomatal density (D), Seed thickness (E) and Trichomes density (D) of *Herpetospermum pedunculosum* (Ser.) Baill. The plant samples were selected from different altitude (2800 m, 3000 m, 3100 m and 3300 m), respectively. Different letters above bars indicate statistically significant differences between different altitudes at *P* < 0.05 according to Duncan’s multiple range Test. Significant differences *P* < 0.01 are indicated two asterisks.
