# Supplementary material for: The adaptation strategies of Herpetospermum pedunculosum (Ser.) Baill at altitude gradient of the Tibetan plateau by physiological and metabolomic methods
Source: BMC Genomics. 2019 Jun 3;20:451. doi: 10.1186/s12864-019-5778-y (PMC6547600; doi:10.1186/s12864-019-5778-y)
Supplement: Supplementary file 2 — Table S1. Effects of altitude gradient on net photosynthetic rate (Pn), stomatal conductance (Gs), intercellular CO2 concentration (Ci), transpiration (Tr) and the maximum efficiency of PSII (Fv/Fm) in leaves of Herpetospermum pedunculosum (Ser.) Baill. (DOCX 1630 kb) [file 12864_2019_5778_MOESM2_ESM.docx]

**Table S1** Effects of altitude gradient on net photosynthetic rate (*P*n), stomatal conductance (*G*s), intercellular CO_2_ concentration (*C*i), transpiration (*T*r) and the maximum efficiency of PSII (*F*v/*F*m) in leaves of *Herpetospermum pedunculosum* (Ser.) Baill. The plant samples were selected from different altitude (2800 m, 3000 m, 3100 m and 3300 m), respectively. Values are means ± SE (*n* = 4). Within a column, values followed by different letters are significantly different at *P* < 0.05 according to Duncan’s multiple range test.

| Altitude  (m) | *Pn* (µmol · m^-2^ · s^-1^) | *Gs* (mmol · m^-2^ · s^-1^) | *Ci*  (µmol CO_2_ · mol^-1^) | *Tr*  (mmol · m^-2^ · s^-1^) | *Fv*/*Fm* |
| --- | --- | --- | --- | --- | --- |
| 2800 | 5.06±0.620^b^ | 0.043±0.004^b^ | 238.94±11.228^a^ | 1.23±0.066^a^ | 0.78±0.006^a^ |
| 3000 | 5.91±0.705^ab^ | 0.063±0.004^a^ | 227.63±27.794^a^ | 1.09±0.221^a^ | 0.79±0.007^a^ |
| 3100 | 7.20±1.004^a^ | 0.063±0.005^a^ | 189.31±39.823^b^ | 0.96±0.168^a^ | 0.80±0.005^a^ |
| 3300 | 4.70±0.374^b^ | 0.041±0.007^b^ | 171.00±25.490^b^ | 0.95±0.168^a^ | 0.79±0.006^a^ |
