# Supplementary material for: The adaptation strategies of Herpetospermum pedunculosum (Ser.) Baill at altitude gradient of the Tibetan plateau by physiological and metabolomic methods
Source: BMC Genomics. 2019 Jun 3;20:451. doi: 10.1186/s12864-019-5778-y (PMC6547600; doi:10.1186/s12864-019-5778-y)
Supplement: Supplementary file 3 — Figure S2. Orthogonal projections to latent structures discriminant analysis (OPLS-DA) score scatter plot derived from the GC-MS data set for leaves of Herpetospermum pedunculosum (Ser.) Baill. (DOCX 98 kb) [file 12864_2019_5778_MOESM3_ESM.docx]

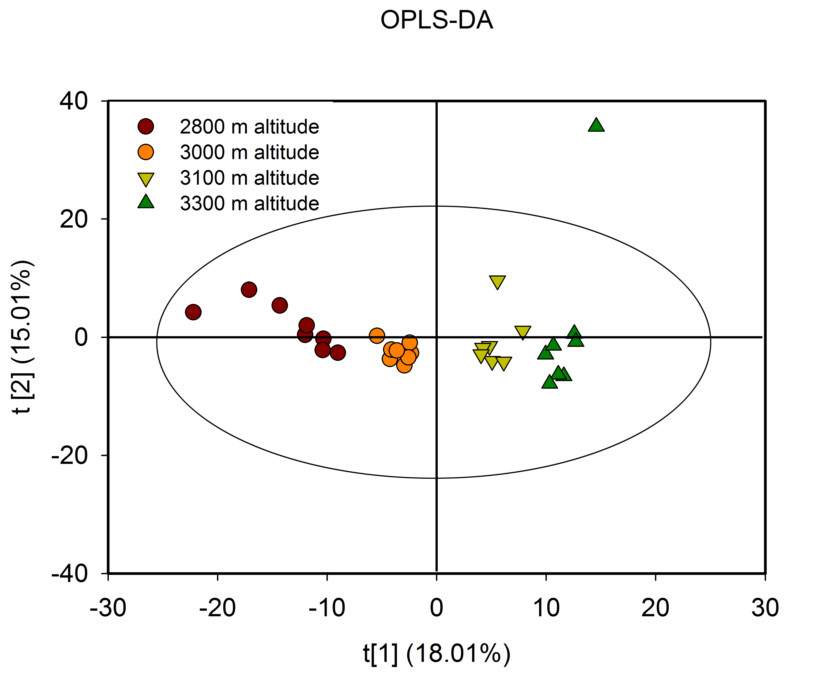


**Figure S2** Orthogonal projections to latent structures discriminant analysis (OPLS-DA) score scatter plot derived from the GC-MS data set for leaves of *Herpetospermum pedunculosum* (Ser.) Baill. Each point represents the metabolic profile of eight replicates of samples collected at four altitudes. Brown circle indicted samples collected in 2800 m altitude; orange circle indicted samples collected in 3000 m altitude; yellow down-triangle indicated samples collected in 3100 m altitude; D: green up-triangle indicated samples collected in 3300 m altitude.
