# Supplementary material for: The adaptation strategies of Herpetospermum pedunculosum (Ser.) Baill at altitude gradient of the Tibetan plateau by physiological and metabolomic methods
Source: BMC Genomics. 2019 Jun 3;20:451. doi: 10.1186/s12864-019-5778-y (PMC6547600; doi:10.1186/s12864-019-5778-y)
Supplement: Supplementary file 4 — Table S2. The relative contents of the identified metabolites in leaves of Herpetospermum pedunculosum (Ser.) Baill. (DOC 97 kb) [file 12864_2019_5778_MOESM4_ESM.doc]

**Table S2**  The relative contents of the identified metabolites in leaves of *Herpetospermum pedunculosum* (Ser.) Baill. normalized to the contents found in 2800 m. AⅡ/ AⅠ: The relative contents of the identified metabolites between 3000 m and 2800 m; AⅢ/ AⅠ: The relative contents of the identified metabolites between 3100 m and 2800 m; AⅣ/ AⅠ: The relative contents of the identified metabolites between 3300 m and 2800 m. + indicates metabolites found in 3000 m; ○ indicates metabolites found in 3100 m; ***** indicates metabolites found in 3300 m. - indicates no metabolites found at different altitude.

| **Group** | | **AⅠ** | **AⅡ/ AⅠ** | **AⅢ/ AⅠ** | **AⅣ/ AⅠ** |
| --- | --- | --- | --- | --- | --- |
| **Carbohydrates** | | | | | |
| Saccharic acid | **+ ○** ***** | 1.00 | 6.73 | 4.24 | 6.98 |
| Glucose-1-phosphate | **+ ○ *** | 1.00 | 6.65 | 4.01 | 7.72 |
| Levoglucosan | **+ ○ *** | 1.00 | 40.09 | 11.54 | 21.17 |
| sucrose | **○** | 1.00 | - | 0.10 | - |
| D-Altrose | **○ *** | 1.00 | - | 8.24 | 3.34 |
| Threitol | **+ ○** | 1.00 | 0.63 | 0.62 | - |
| 1-Kestose | **○ *** | 1.00 | - | 3.99 | 2.05 |
| glycerol | **○ *** | 1.00 | - | 2.54 | 3.95 |
| D-Arabitol | **○ *** | 1.00 | - | 1.75 | 1.87 |
| raffinose | **○ *** | 1.00 | - | 0.42 | 0.47 |
| Galactonic acid | **+ ○** | 1.00 | 0.63 | 0.63 | - |
| glucuronic acid | **+** | 1.00 | 4.46 | - | - |
| Melezitose | **○** | 1.00 | - | 11.23 | - |
| Digalacturonic acid | **+ ○ *** | 1.00 | 0.31 | 0.19 | 0.21 |
| Diglycerol | **+ ○** | 1.00 | 14.32 | 12.59 | - |
| ribulose-5-phosphate | **+** | 1.00 | 0.31 | - | - |
| maltitol | **○** | 1.00 | - | 0.39 | - |
| L-Gulonolactone | **○** | 1.00 | - | 1.84 | - |
| Tagatose | **○ *** | 1.00 | - | 0.48 | 0.24 |
| **Amino acids** | | | | | |
| L-homoserine | **+** | 1.00 | 61.46 | - | - |
| 4-aminobutyric acid | **○** | 1.00 | - | 1.68 | - |
| threonine | **○ *** | 1.00 | - | 18.68 | 14.59 |
| aspartic acid | **+ ○ *** | 1.00 | 6.06 | 7.96 | 14.46 |
| N-Acetyl-L-aspartic acid |  | 1.00 | - | - | - |
| serine | ***** | 1.00 | - | - | 4.89 |
| proline | **○ *** | 1.00 | - | 7.80 | 2.79 |
| 2,6-Diaminopimelic acid | **+ ○** | 1.00 | 0.05 | 0.05 | - |
| oxoproline | **+ *** | 1.00 | 0.28 | - | 0.32 |
| carbamoyl-aspartic acid | **+** | 1.00 | 0.05 | - | - |
| 5-hydroxytryptophan | **○** | 1.00 | - | 0.30 | - |
| leucine | **○** | 1.00 | - | 8.65 | - |
| alanine | **○** | 1.00 | - | 2.24 | - |
| phenylalanine | **○** | 1.00 | - | 4.10 | - |
| **Organic acids** | | | | | |
| citric acid | **○ *** | 1.00 | - | - | - |
| 3,4-dihydroxycinnamic acid | **+** | 1.00 | 2.27 | - | - |
| Threonic acid | **+ *** | 1.00 | 1.92 | - | 1.42 |
| beta-dydroxypyruvate | **○** | 1.00 | - | 2.11 | - |
| malonic acid | **+** | 1.00 | 2.47 | - | - |
| alpha-ketoglutaric acid | **○** | 1.00 | - | 0.28 | - |
| oxalic acid | ***** | 1.00 | - | - | 3.39 |
| **Lipid components** | | | | | |
| Ethanolamine | ***** | 1.00 | - | - | 2.58 |
| 3-Hexenedioic acid | ***** | 1.00 | - | - | 12.39 |
| **Polyamine** | | | | | |
| spermidine | ***** | 1.00 | - | - | 4.73 |
| **Secondary metabolism** | | | | | |
| Tricetin | **+ ○** | 1.00 | 83.85 | 23.84 | - |
| Neohesperidin | **○** | 1.00 | - | 4.96 | - |
| caffeic acid | **+** | 1.00 | 5.55 | - | - |
| 1-Hydroxyanthraquinone | ***** | 1.00 | - | - | 2.66 |
| **Others** | | | | | |
| putrescine | **○ *** | 1.00 | - | 3.29 | 2.58 |
| 1,2,4-Benzenetriol | ***** | 1.00 | - | - | 6.73 |
| Dodecanol | ***** | 1.00 | - | - | 4.69 |
