# Supplementary material for: The adaptation strategies of Herpetospermum pedunculosum (Ser.) Baill at altitude gradient of the Tibetan plateau by physiological and metabolomic methods
Source: BMC Genomics. 2019 Jun 3;20:451. doi: 10.1186/s12864-019-5778-y (PMC6547600; doi:10.1186/s12864-019-5778-y)
Supplement: Supplementary file 6 — Figure S3. Changes in metabolite concentrations in leaves of Herpetospermum caudigerum Wall. at four altitudes including 2800 m, 3000 m, 3100 m and 3300 m (A, samples collected in 2800 m; B, samples collected in 3000 m; C, samples collected in 3100 m; D, samples collected in 3300 m). B/A: The ratio of the B concentration to the A concentration is shown by red colored bars. (DOC 1120 kb) [file 12864_2019_5778_MOESM6_ESM.doc]

*****

*****

*****

*****

*****

**Figure S3** Changes in metabolite concentrations in leaves of *Herpetospermum caudigerum* Wall. at four altitudes including 2800 m, 3000 m, 3100 m and 3300 m (A, samples collected in 2800 m; B, samples collected in 3000 m; C, samples collected in 3100 m; D, samples collected in 3300 m). B/A: The ratio of the B concentration to the A concentration is shown by red colored bars. Similarly, C/A: C/A concentration ratios for metabolites whose mean concentrations changed between C and A indicted by green bars. D/A: D/A concentration ratios for metabolites whose mean concentrations changed between D and A are indicted by blue bars. Bold indicates metabolites found in B, C and D. ***** indicted the metabolites having significant change.
