# Supplementary material for: The adaptation strategies of Herpetospermum pedunculosum (Ser.) Baill at altitude gradient of the Tibetan plateau by physiological and metabolomic methods
Source: BMC Genomics. 2019 Jun 3;20:451. doi: 10.1186/s12864-019-5778-y (PMC6547600; doi:10.1186/s12864-019-5778-y)
Supplement: Supplementary file 7 — Figure S4. Classification of metabolites according to different altitudes. (DOCX 100 kb) [file 12864_2019_5778_MOESM7_ESM.docx]

**113/33**

**D/A**

**C/A**

**A**

**103/34**

**134/21**

B/A

**Figure S4** Classification of metabolites according to different altitudes. The numbers on the slash arrows indicated the number of all identified metabolites in two altitudes and the numbers beneath the slash indicated the number of different identified metabolites in two altitudes. A, samples collected in 2800 m; B, samples collected in 3000 m; C, samples collected in 3100 m; D, samples collected in 3300 m.
